# Evaluating the Oral-Health-Related Quality of Life of Oral Submucous Fibrosis Patients before and after Treatment Using the OHIP-14 Tool

**DOI:** 10.3390/ijerph19031821

**Published:** 2022-02-05

**Authors:** Abdul Bari Memon, Aneela Atta Ur Rahman, Kashif Ali Channar, Muhammad Sohail Zafar, Naresh Kumar

**Affiliations:** 1 Medical Research Centre, Liaquat University of Medical and Health Sciences, Jamshoro 76090, Pakistan; drabmemon@smbbmu.edu.pk (A.B.M.); aneela.atta@lumhs.edu.pk (A.A.U.R.); 2 Department of Community Dentistry, Bibi Aseefa Dental College, Shaheed Mohtarama Benazir Bhutto Medical University, Larkana 77150, Pakistan; 3 Faculty of Community Medicine and Public Health Sciences, Shaheed Mohtarama Benazir Bhutto Medical University, Larkana 77150, Pakistan; 4 Department of Oral and Maxillofacial Surgery, Institute of Dentistry, Liaquat University of Medical and Health Sciences, Jamshoro 76090, Pakistan; kashif.channar@lumhs.edu.pk; 5 Department of Restorative Dentistry, College of Dentistry, Taibah University, Al Madinah, Al Munawwarah 41311, Saudi Arabia; mzafar@taibahu.edu.sa; 6 Department of Dental Materials, Islamic International Dental College, Riphah International University, Islamabad 44000, Pakistan; 7 Department of Science of Dental Materials, Ishrat Ul Ebad Khan Institute of Oral Health Sciences, Dow University of Health Sciences, Karachi 74200, Pakistan

**Keywords:** oral submucous fibrosis, oral health, oral potentially malignant disorders, oral cancer, quality of life

## Abstract

The objective of the present study was to assess the oral-health-related quality of life (OHRQoL) of oral submucous fibrosis (OSMF) patients before and after standard treatment. A convenient sampling technique was used to recruit the clinically diagnosed patients of OSMF (n = 130). Based on the medical treatment, the patients were randomly divided into two study groups (group A and B). The group A patients received submucosal intralesional injections of dexamethasone (2 mL; 4 gm/mL), while group B patients received hyaluronidase (1500 IU). Both the group A and B patients received respective medical therapy biweekly for a period of five weeks. At the follow up visit (6 months), the impact of treatment on OHRQoL was assessed using the Oral Health Impact Profile-14 (OHIP-14). Data were analyzed by a chi-square test for quantitative variables and an independent *t*-test for qualitative variables. The comparison of all clinical parameters before and after treatment was performed by a paired *t*-test. The results after treatment showed that there was a significant improvement in all domains of OHIP-14 (*p* = 0.001) except psychological disability (*p* = 0.243). In addition, the OHRQoL of patients was significantly improved following the treatment.

## 1. Introduction

Oral submucous fibrosis (OSMF) is a chronic, progressive and irreversible oral potentially malignant disorder that causes the blanching, stiffening and fibrosis of oral mucosa of different areas including lips, buccal mucosa, tongue, soft palate, and anterior pillar of the fauces [1,2]. The most characteristic feature of OSMF is the blanching (marble-like appearance) of buccal mucosa by impairing the local blood vessels [2]. Epidemiological findings indicate that the OSMF is highly prevalent in Southeast Asian countries, particularly in the Indo-Pak sub-continent [2,3,4]. OSMF remains a great dental public health concern due to its significant transformation rate (7% to 13%) to oral cancers [5]. The pathogenesis of the disease is not fully understood; however, its etiology is believed to be multi-factorial. The possible causative factors of OSMF are deficiencies of essential vitamins, zinc, iron, and the presence of capsaicin present in chilies [6,7,8]. Moreover, epidemiological studies have clearly documented that areca nut is the most common causative agent for developing OSMF [1,3]. The clinical presentation of OSMF depends on the disease stage and may include impaired mouth movement, marked rigidity, atrophy of muscle fiber, intolerance to eating hot and spicy food, inability to open mouth, burning sensation of oral cavity, recurrent oral ulceration, and reduced mobility of soft palate, which ultimately leads to further rigidity and disability in the mouth opening [9,10].

The management of OSMF aims to cure the inability to open the mouth and the burning sensation that is caused by an intolerance to spicy food, inhibiting disease progression and decreasing the risks of the malignant transformation. Evidence from the literature [11,12,13] recommended that medical treatment included placental extracts, hyaluronidase, steroids, interferon gamma, pentoxifilline, and lycopene, while physical treatment aims to influence tissue remodeling by different methods, namely physiotherapy, exercises and splints to improve mouth opening. Furthermore, intralesional injections of various therapeutic agents, such as hyaluronidase (HD), dexamethasone (DM), triamcinolone, and placental extract, have demonstrated promising outcomes in terms of pain reduction and improvement in mouth opening. Intralesional injections have demonstrated no or minimal adverse effects due to minimal systemic absorption. Moreover, while treating OSMF patients, a variety of drug regimens have been employed, and each drug exhibited a different mechanism of action [14]. For example, steroids play a key role in the prevention of fibrosis due to their anti-fibrotic action. This mechanism of action leads to a decrease in the proliferation of fibroblasts and deposition of collagen, consequently providing relief from symptoms. In addition, anti-inflammatory role of steroids is well-evident [15]. Hyaluronidase disintegrates and dissolves the fibrous bands, which provides relief from symptoms. It also causes the breakdown of hyaluronic acid, which eventually decreases the viscosity of the intracellular substance [16].

Oral-health-related quality of life (OHRQoL) can be defined as an assessment of individuals regarding the oral health and how functional, psychological, social factors, pain, or discomfort affect the well-being of an individual [17]. Considering that oral diseases commonly affect quality of life (QoL), OHRQoL is a useful research tool that has been frequently used by oral researchers [18,19]. Any ailment that can obtrude in the performance of routine dental tasks may also have detrimental consequence on general QoL. Therefore, after many analyses of the influence of oral diseases on different aspects of life, the concept of OHRQoL was developed [20]. Quality of life may be disturbed due to oral diseases, which may affect the general well-being and everyday life activities of patients [21,22]. However, the published literature contains studies that mainly emphasized the management of OSMF, with little emphasis on the improvement of QoL; these studied drugs have made great contributions to maintaining health [23]. Drug therapy forms an inseparable part of OSMF management, which, apart from increasing mouth opening, and decreasing pain and a burning sensation, relieves patients of severe fibrotic changes which may ultimately improve the QoL of patients [24]. Therefore, the aim of the present study was to evaluate the impact of the intralesional administration of HD and DM on patients’ OHRQoL.

## 2. Methods

### 2.1. Study Participants

This prospective study was conducted in a tertiary care hospital of the Laiquat University of Medical and Health Sciences, Jamshoro, Pakistan. Ethical permission was sought from the Ethical Review Committee of the University (Reference: NO.LUMHS/REC/-640;26/12/17). The clinically diagnosed patients of OSMF were recruited using a convenient sampling technique (n = 130). The Openepi online calculator was used to calculate the sample size. The study [25] that compared the sub-mucosal injections of HD and DM in patients with histo-pathologically confirmed OSMF was used to compute the sample size. The study reported a mean reduction in pain, while opening mouth in the HD group was 1.00 ± 1.69, compared to DM group (0.33 ± 0.72); the sample size calculated at 95% confidence interval, considering power as 80%, which is a total of 118 patients, with 59 patients allocated to each group with a ratio of 1:1. To account for the non-response and loss to follow-up, the sample size was increased by 10%, thus the final sample size was 130. The inclusion criteria were patients with clinically diagnosed for OSMF, inter-incisal mouth opening (IIMO) between 15–35 mm, patients of either gender, patients over 18 years of age, patients not receiving any treatment for OSMF, patients agreeing to visit regularly for follow-up in accordance with the treatment protocol. The exclusion criteria were: patients with bleeding dyscrasias; patients with other causes of limited mouth opening (temporo-mandibular problems or pericoronitis, scleroderma, burns); patients with drug allergies or hypersensitivity to HD, DM or lignocaine; patients with any other mucosal disease (such as aphthous ulcers, leukoplakia, erythroplakia, oral squamous cell carcinoma) or skin lesions associated with oral lesions; pregnant and lactating women; patients with any systemic disease; and patients not willing to give up habits of chewing tobacco and betel nuts. A written informed consent was taken from the participants before the commencement of the study. Demographic information was recorded using the designed questionnaire. A detailed history of the disease and associated factors, such as habits and kind of areca nut used, were recorded.

### 2.2. Clinical Examination

Two clinicians individually evaluated the patients for the confirmation of diagnosis of OSMF. The patients were enrolled after a consensus was achieved by both clinicians, who each had more than five years of clinical experience in the field of oral and maxillofacial surgery. A total of 130 patients were randomly allocated in two study groups (Group A and B). In group A (n = 65), sub-mucosal injection of Decadron (OBS Pakistan Pvt. Ltd., Karachi, Pakistan) (containing DM 2 mL (4 mg/mL) and 1 mL of 2% lignocaine with adrenaline (1:100,000) (Huons Co., Ltd., Seongnam, Korea) was administered biweekly on the buccal mucosa of both sides for a period of 5 weeks. In group B (n = 65), sub-mucosal injection of Hylase containing HD 1500 IU (Wockhardt UK Ltd., Wrexham, UK), diluted with 2 mL of water for injection (Macter International Limited, Karachi, Pakistan) and 1 mL of 2% lignocaine with adrenaline (1:100,000) (Huons Co., Ltd., Korea), was administered biweekly on the buccal mucosa of both sides for a period of 5 weeks. Out of 130 patients, nine patients from each group did not attend the clinic at the 6-month follow up. The remaining 112 patients (n = 56 for each group) were included.

### 2.3. Oral-Health-Related Quality of Life (OHRQoL)

The OHRQoL was measured by a shorter version of Oral Health Impact Profile-14 (OHIP-14) [26]. Briefly, the OHIP-14 questionnaire comprised 14 items and covered seven domains: functional limitations, physical disability, psychological disability, physical pain, psychological discomfort, social disability, and handicap. A 5-point scale (0 for never to 4 for very often) was used to calculate the score for each item. The total score of OHIP-14 is in the range of 0 to 56. The data of all patients were recorded in the questionnaire before the commencement of treatment and after six months of treatment.

### 2.4. Statistical Analysis

Data were recorded on a questionnaire and analyzed using SPSS version-16 (IBM Corp., Armonk, NY, USA). The qualitative variables (such as gender, age categories, kind of chewing habit and status of quality of life before and after treatment) were highlighted as frequency and percentage, whereas the quantitative variable (i.e., age) was presented as mean ± SD. Demographic characteristics, including age groups, gender, and kind of chewing habits, were compared between the two groups using chi-square statistics. Comparison of QoL pre and post treatment was performed by paired *t*-test. For the purpose of inferential statistics, the *p*-value < 0.05 was considered significant.

## 3. Results

### 3.1. Demographic Characteristics

There were no participants who reported any complications or adverse effects associated with the intralesional administration of the drug; however, a number of patients complained of pain and swelling after taking intralesional injections, which subsided by itself. The age range of patients was from 18 years to 65 years (mean: 41.0 ± 11.2 years). There was no significant difference in the mean age (*p* = 0.186) of group A patients (42.4 ± 11.6 years) and group B patients (39.6 ± 10.8 years). The male-to-female ratio was 1.9:1, corresponding to 74 (66%) male and 36 (34%) female participants. There was no significant difference in the genders of both treatment groups (*p* = 0.425) (Table 1).

**Figure 1:**
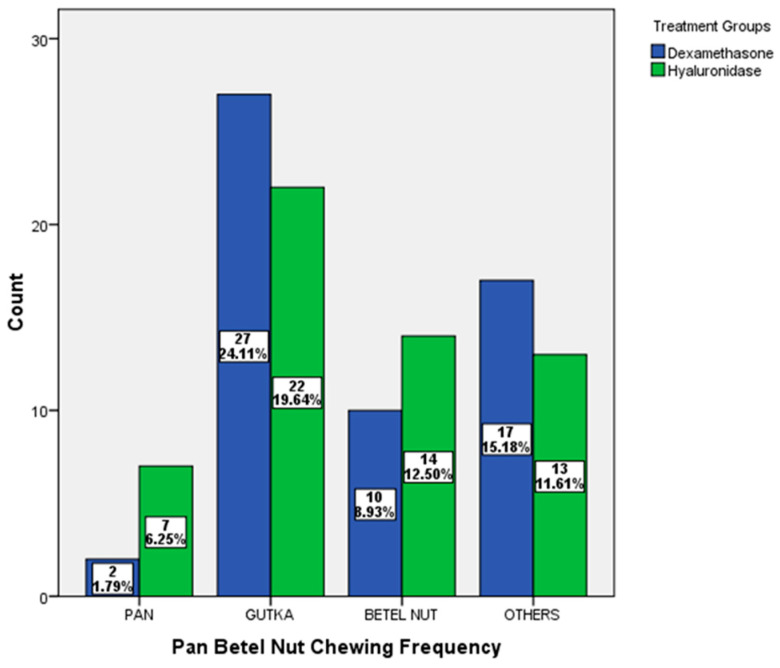
Distribution of chewing habits according to the treatment groups.

All patients (100%) habitually chewed pan, gutka, and betel nut, both alone and together. Gutka chewing was the predominant habit in both treatment groups. Both groups showed a statistically insignificant difference (*p* = 0.213) with respect to deleterious habits (Figure 1).

### 3.2. Seven Domains of OHIP-14 Items

The OHIP-14 questionnaire was filled before starting the treatment and after 6 months. The responses of OHIP-14 showed that the majority of patients occasionally reported responses in the following categories: difficulty doing usual job (21%), felt tense (21%), bit irritable (22%), worried by dental problems (32%), uncomfortable to eat (33%) and diet unsatisfactory (35%) before treatment, whereas the same responses were occasionally reported after treatment and showed an improvement except for the ‘felt tense’ category (Table 2).

The OHRQoL was compared for all patients before and after treatment. The results showed that, apart from psychological disability (*p* = 0.243), there was a significant improvement in all domains after treatment. Patients reported significant improvements in the domains of functional limitation (*p* = 0.001), physical pain (*p* = 0.001), social disability (*p* = 0.001), psychological discomfort (*p* = 0.001), physical disability (*p* = 0.001) and handicap (*p* = 0.001). OHIP scores were improved after treatment in all the domains of QoL (Table 3).

Table 4 highlights the QoL in both treatment groups. There was insignificant difference with regard to QoL in both treatment groups (*p* < 0.05).

## 4. Discussion

The present institutional-based research study evaluated the effects of the localized delivery of DM and HD on the OHRQoL of patients suffering from OSMF. For this purpose, the intralesional injections were administered to attain a high concentration at the local site of action, with minimal systemic absorption. An intralesional administration route was selected for its benefits, such as no or minimal side effects compared to systemic administration, cost effectiveness, and no need for patient consent [27]. In addition, it plays a significant role in comforting the patient by relieving symptoms. Many treatment modalities, such as medical, surgical and physiotherapy treatments, have been advocated to overcome the symptoms of OSMF patients; however, no definitive treatment is currently available. Moreover, each treatment modality has its own limitations [28]. In the present study, the proportion of males (66%) is greater than females (34%), which is in agreement with previous studies [29,30]. On the contrary, a few studies [31,32] reported more involvement of females. Males use smokeless tobacco products more often than females; therefore, OMSF is more prevalent in males, and this is reflected in the present study [33,34].

When considering the habit of chewing areca nut, most of the study participants reported a history of using chewed gutka (43.8%). These findings are in agreement with previous studies [35,36,37]. In our study, all patients had a habit of chewing at least one smokeless tobacco product, and the consumption of such products was identified as the major etiological factor. The age of the majority of OSMF patients was 31–50 years (61%), which is in agreement with the previous study [38]. However, some previous studies [39,40] reported those in their 30s as the most common age group. It may be attributed to the fact that the consumption of smokeless tobacco between 20–30 years of age was comparatively low due to the restricted sale of such agents, or the appearance of symptoms was delayed until the age of 30 years due to active body immunity.

In the baseline questionnaire before the treatment regarding the QoL, it was found that the greatest impact was on the physical pain domain, where patients felt ‘painful aching’ fairly often (40%) and very often (29.5%) followed by ‘uncomfortable to eat any food’ as occasionally (34%). This was followed by the physical discomfort domain, where patients occasionally felt that their ‘diet was unsatisfactory’ (44%). These results are similar to the previous study [41] and clearly suggested that patients’ daily activity might be compromised due to the disease. When comparing the QoL before and after 6 months of treatment, most of the items showed improvements in the handicap domain, with patients giving the response of never in ‘totally unable to function’ (89%), ‘life less satisfying’ (66%), followed by functional limitation domains where the highest impact was the response of never in ‘trouble pronouncing any word’ (82%) and ‘sense of taste’ has worsened (48%); those in the social disability domain responded as never for ‘difficulty doing usual jobs’ (67%). There was improvement in the physical pain domain, responding with occasionally in ‘painful aching’ and hardly ever in ‘uncomfortable to eat’: 53% and 45.5%, respectively, and the psychological discomfort domain responded as hardly ever in ‘worried by dental problem’ and ‘felt tense’: 55% and 51%, respectively, after treatment. The QoL of patients improved after treatment, which is in agreement with the results of Rimal and Shrestha [41]. The paired mean differences showed significant improvements in all domains except psychological disability; this could be due to patients already worried and afraid of dental treatment, and feeling tense because of fear of injections. There was high OHIP score before treatment in this study.

This clearly shows that OHRQoL was impaired. There was a compromise in their daily activities such as a sense of bad taste, oral pain, uncomfortable eating, worrying about a dental problem, unsatisfactory dietary habits, feeling slight support, job difficulties, and feeling as though life is less satisfying due to the disease. The QoL of these patients was significantly improved following the treatment.

When comparing the mean domains treated with DM and HD, all of the domains showed statistically insignificant results, indicating no difference between either drug on QoL, which suggests that both drugs are equally effective in terms of QoL (Table 4). Moreover, to the best of the authors’ knowledge, not a single study has compared the impact of each drug alone on the QoL of OSMF patients.

There are certain limitations of the present study: only clinical methods were applied during the clinical dental examination, and a biopsy was not performed. The definitive diagnosis of OSMF is based on histology, although clinical diagnosis can be readily obtained based on clinical exam/symptoms, combined with a known use of betel nut. Histologically, biopsy often reveals an inflamed and atrophied oral mucosa, with a fibrosis of the submucosa and may lead to further scar formation, worsening symptoms [7,42,43,44]. Moreover, it is an invasive, time-consuming procedure and may cause potential psychological trauma to patients. Considering the institutional ethical board guidelines and objective of this study, a biopsy and histopathological analysis were not performed, while the diagnosis of OSMF was confirmed through a clinical examination independently performed by two experienced clinicians. Furthermore, this may be considered as one of the limitations of this study, and we recommend that diagnosis is confirmed through a histopathological analysis in future research.

Most of the patients in stage 1 are symptomless and reluctant to receive treatment, while stage 4 patients usually require surgical treatment. Therefore, the present study investigated two functional stages (M-2 and M-3). There may have been operator-related bias while assessing the clinical parameters and completing the questionnaire. There was no control group as, due to social and financial constraints, patients were unwilling to participate and cooperate without having any active treatment. The limited sample size and loss to follow up may influence the study, and psychometric properties of the scale may vary in a larger subset of population. Oral screening programs may be arranged for the early detection of lesions to improve QoL. It is recommended that future studies include questionnaires designed specifically to evaluate how well those treated for OSMF can perform common functions. It is further recommended that multi-centered, long-term, follow-up studies with increased sample sizes may be planned to assess any improvement in OHRQoL in patients treated for OSMF.

## 5. Conclusions

There was a significant improvement after treatment in all domains of OHIP-14, except psychological disability. In addition, the OHRQoL of these patients was significantly improved following the treatment. The intralesional administration of HD and DM was equally efficient in reducing the pain and intensity of BS in oral sub-mucous fibrosis patients.

## Figures and Tables

**Table 1:** Descriptive statistics of demographic data of patients who participated in the current study.

Variables	Categories	Group A	Group B	*p*-Value
Age	Mean ± SD	42.4 ± 11.6	39.6 ± 10.8	0.186
Gender	Male (%)	39 (69.6)	35 (62.5)	0.425
	Female (%)	17 (30.4)	21 (37.5)	

**Table 2:** Descriptive statistics of quality of life among OSMF patients before and after treatment.

	Never		Hardly Ever		Occasionally		Fairly Often		Very Often	
	N	(%)	N	(%)	N	(%)	N	(%)	N	(%)
**Functional Limitations**										
**Trouble pronouncing any word**										
Before treatment	**68**	**60.7**	27	24.1	15	13.1	2	1.8		
After treatment	**92**	**82.1**	18	16.1	1	0.9	1	0.9		
**Sense of taste has worsened**										
Before treatment	43	38.4	**53**	**47.3**	12	10.7	3	2.7	1	0.9
After treatment	**54**	**48.2**	40	35.7	17	15.2	1	0.9		
**Physical Pain**										
**Painful aching**										
Before treatment	1	0.9	11	9.8	22	19.6	**45**	**40.2**	33	29.5
After treatment	6	5.4	20	17.9	**59**	**52.7**	26	23.2	1	0.9
**Uncomfortable to eat any food**										
Before treatment	7	6.2	22	19.6	**38**	**33.9**	35	31.2	10	8.9
After treatment	14	12.5	**51**	**45.5**	39	34.8	8	7.1		
**Psychological Discomfort**										
**Worried by dental problem**										
Before treatment	14	12.5	**38**	**33.9**	34	30.4	23	20.5	3	2.7
After treatment	19	17.0	**62**	**55.4**	30	26.8	1	0.9		
**Felt tense**										
Before treatment	29	25.9	**45**	**40.2**	22	19.6	14	12.5	2	1.8
After treatment	33	29.5	**57**	**50.9**	22	19.6				
**Physical Discomfort**										
**Diet unsatisfactory**										
Before treatment	14	12.5	36	32.1	**39**	**34.8**	17	15.2	6	5.4
After treatment	28	25.0	**63**	**56.2**	18	16.1	3	2.7		
**Interrupted meal**										
Before treatment	39	34.8	**42**	**37.5**	22	19.6	8	7.1	1	0.9
After treatment	40	35.7	**62**	**55.4**	10	8.9				
**Psychological Disability**										
**Difficult to relax**										
Before treatment	36	32.1	**48**	**42.9**	21	18.8	7	6.2		
After treatment	40	35.7	**61**	**54.5**	8	7.1	2	1.8	1	0.9
**Bit Embraced**										
Before treatment	42	37.5	**47**	**42.0**	15	13.4	7	6.2	1	0.9
After treatment	48	42.9	**56**	**50.0**	8	7.1				
**Social Disability**										
**Bit irritable**										
Before treatment	33	29.5	**40**	**35.7**	25	22.3	13	11.6	1	0.9
After treatment	58	51.8	**41**	**36.6**	9	8.0	4	3.6		
**Difficulty doing usual jobs**										
Before treatment	**52**	**46.4**	34	30.4	24	21.4	2	1.8		
After treatment	**75**	**67.0**	29	25.9	5	4.5	3	2.7		
**Handicap**										
**Life less satisfying**										
Before treatment	**42**	**37.5**	18	16.1	23	20.5	22	19.6	7	6.2
After treatment	**74**	**66.1**	18	16.1	16	14.3	3	2.7	1	0.9
**Totally unable to function**										
Before treatment	**91**	**81.2**	14	12.5	6	5.4	1	0.9		
After treatment	**100**	**89.3**	10	8.9	2	1.8				

Bold represents the majority percentage encountered in each of the fourteen OHIP items.

**Table 3:** Relationship of quality of life among OSMF patients before and after treatment by paired *t*-test.

OHRQoL before and after Treatment						
Dimension	Before Treatment (n = 112)		After Treatment (n = 112)			
	Mean	SD	Mean	SD	Paired Mean Difference	*p*-Value
Functional Limitations	3.37	1.230	2.89	1.051	0.473	0.001
Physical Pain	7.04	1.773	5.33	1.442	1.714	0.001
Psychological Discomfort	4.91	1.966	4.02	1.301	0.893	0.001
Physical Discomfort	4.71	1.748	3.42	1.213	1.286	0.001
Psychological Disability	3.90	1.698	3.70	1.236	0.205	0.243
Social Disability	3.99	1.742	3.06	1.282	0.929	0.001
Handicap	3.67	1.657	2.69	1.014	0.982	0.001
Total OHIP score	31.59	9.420	25.11	5.862	6.482	0.001

*p* < 0.05 significant.

**Table 4:** Comparison of mean score of 7 domains of OHIP-14 item in dexamethasone and hyaluronidase groups.

7 Domains	Functional Limitations	Physical Pain	Psychological Discomfort	Physical Discomfort	Psychological Disability	Social Disability	Handicap	Total OHIP Score
Dexamethasone (n = 56)	2.88 ± 1.08	5.39 ± 1.28	4.18 ± 1.29	3.61 ± 1.24	3.88 ± 1.23	3.00 ± 1.27	2.61 ± 0.96	25.54 ± 5.72
Hyaluronidase (n = 56)	2.91 ± 1.03	5.27 ± 1.59	3.86 ± 1.29	3.23 ± 1.16	3.52 ± 1.221	3.12 ± 1.29	2.77 ± 1.06	24.68 ± 6.01
*p*-value *	0.858	0.649	0.192	0.102	0.127	0.608	0.404	0.442

* *p* value > 0.05 highlights that there was no significant difference in each OHIP item in relation to treatment groups.

## Data Availability

The data presented in this study are available on the request from the corresponding author.
